# Comparing methotrexate monotherapy with methotrexate plus leflunomide combination therapy in psoriatic arthritis: protocol of a randomized, placebo-controlled, double-blind clinical trial (COMPLETE-PsA)

**DOI:** 10.1186/s13063-020-4097-6

**Published:** 2020-02-10

**Authors:** Michelle L. M. Mulder, Johanna E. Vriezekolk, Nathan den Broeder, Elien A. M. Mahler, Philip S. Helliwell, Frank H. J. van den Hoogen, Alfons A. den Broeder, Mark H. Wenink

**Affiliations:** 1 Department of Rheumatology, Sint Maartenskliniek, PO Box 9011, Nijmegen, 6500 GM The Netherlands; 2 Department of Rheumatic Diseases, Radboud University Medical Center, Nijmegen, The Netherlands; 3 Leeds Institute of Rheumatology and Musculoskeletal Medicine, University of Leeds, Leeds, UK

**Keywords:** Psoriatic arthritis, Methotrexate, Leflunomide, Conventional disease-modifying antirheumatic drug (cDMARD), Combination therapy

## Abstract

**Background:**

Both methotrexate (MTX) and leflunomide (LEF) are registered and regularly prescribed as first-line treatments for the use in patients with psoriatic arthritis (PsA) and they are occasionally used in combination. However, evidence about their individual, and especially combined efficacy, in PsA is lacking. The aim of this study is to compare the effectiveness and safety of MTX and LEF combination therapy to MTX monotherapy in patients with PsA.

**Methods:**

COMPLETE-PsA is a randomized, placebo-controlled, double-blind clinical trial. Disease-modifying antirheumatic drug (DMARD)-untreated patients (n = 78) with clinical diagnosis of active (i.e. ≥2 swollen joints) PsA will be randomized 1:1 (stratified for high disease activity, Psoriatic Arthritis Disease Activity Score [PASDAS] ≥ 5.4) to the combination or monotherapy. The intervention group receives MTX 25 mg (oral or subcutaneous) once weekly plus LEF 20 mg daily, and the control group receives the same but with placebo instead of LEF daily. Primary endpoint is between-group difference in PASDAS at 16 weeks, adjusted for baseline PASDAS. Key secondary parameters include between-group comparisons in change in Disease Activity in Psoriatic Arthritis (DAPSA) score, skin score, enthesitis score, dactylitis score, and swollen/tender joint count, as well as the proportion of patients fulfilling minimal disease activity (MDA), American College of Rheumatology (ACR) 20/50/70 response criteria at week 16. Furthermore, safety, function and quality of life (Health Assessment Questionnaire [HAQ], Psoriatic Arthritic Impact of Disease [PSAID], Short Form 12 [SF-12]) will be assessed.

**Discussion:**

This is, to our knowledge, the first randomized, placebo-controlled, double-blind clinical trial assessing the effectiveness of MTX and LEF combination therapy in patients with PsA. The study will provide important information for treatment strategies and treatment recommendations.

**Trial registration:**

Dutch Trial Register NTR7632 (3 December 2018). CMO NL66544.091.18 (19 November 2018).

## Background

Psoriatic arthritis (PsA) is a chronic inflammatory disease of joints and entheses that occurs in up to 30% of patients with psoriasis that leads to pain, stiffness, and loss of function, and is frequently associated with an additional negative impact on quality of life [1, 2]. It is a heterogeneous disease, with involvement of the joints, entheses, spine, skin, and nails. Besides the heterogeneity of the disease, the lack of high-quality clinical trials assessing the efficacy of traditional disease-modifying therapies is a major challenge in routine clinical practice [3].

For the treatment of peripheral arthritis in PsA, the European League Against Rheumatism (EULAR) and the Group for Research and Assessment of Psoriasis and Psoriatic Arthritis (GRAPPA) both recommend first-line treatment with conventional disease-modifying antirheumatic drugs (cDMARDs) [4, 5]. cDMARDs that are recommended for the early management of PsA are methotrexate (MTX), leflunomide (LEF), cyclosporine, and sulfasalazine. In case of cDMARD treatment failure, the EULAR recommends trying a second cDMARD or switching to tumor necrosis factor alpha inhibitors (TNFi), which are part of the family of biological DMARDs (bDMARDs), depending on the absence or presence of adverse prognostic factors, respectively. In contrast, the American College of Rheumatology recommends treatment with a TNFi over a cDMARD in patients with treatment-naive and active PsA, but remarks that a cDMARD may be considered in some situations (e.g., mild disease) [6].

Although both the GRAPPA and EULAR recommend to start with a cDMARD as first-line therapy, there is limited insight about the efficacy of cDMARDs, and especially their combination, in PsA. To date, almost all the high-quality clinical PsA trials that have been performed, investigate the efficacy of bDMARDs instead of cDMARDs. However, bDMARDs have several drawbacks. They are in most countries only reimbursed after the failure of one or more cDMARDs, not recommended as first-line therapy, not available in oral form, more expensive, and patients are more prone to serious infections compared with cDMARDs. Considering the above, the timely availability of bDMARDs is limited in many situations and countries. Therefore, there is a need for high-quality trials that assess the efficacy of (combination of) cDMARDs in PsA. EULAR also acknowledged the importance of this topic by adding ‘assessing efficacy and safety of combinations of conventional DMARDs (cDMARDs) compared with cDMARD monotherapy’ to their research agenda in 2016 [4].

Of the different cDMARDs, MTX is the one that is most widely used as a first-choice treatment regimen for patients with PsA. It is registered by the European Medicines Agency (EMA) for PsA and was also used as starting drug in the only randomized trial assessing the effect of implementing a treat-to-target treatment strategy in PsA (the TICOPA trial) [7]. MTX is an antimetabolite of the antifolate type, inhibiting purine synthesis, and its efficacy to reduce disease activity, pain, and limitations in rheumatoid arthritis is well established [8]. This is in contrast to PsA, where only low-quality evidence on the effectiveness of (low-dose, 15 mg or less) MTX is available from eight studies, that shows a possible and only very small effect of MTX on disease activity [9]. Unfortunately, the only randomized and placebo-controlled trial that was judged as to be of low risk of bias, failed to show evidence that (low-dose) MTX monotherapy improves synovitis in PsA, when compared to placebo [10].

The other cDMARD with some available evidence on its effectiveness in PsA, and that is also approved by the EMA, is LEF. LEF is a pyrimidine synthesis inhibitor that works by inhibiting dihydroorotate dehydrogenase [11]. A recent meta-analysis states that LEF seems a safe and effective treatment option in PsA [12]. However, only one of the included studies was a randomized and placebo-controlled trial [13]. They concluded that LEF was superior to placebo, with a significantly higher proportion of patients improving in Psoriatic Arthritis Response Criteria (PsARC) (59% vs 30%, respectively). Although the treatment response was larger in patients using LEF, the effect sizes were relatively small. So in conclusion, MTX and LEF seem effective in PsA, but effects are modest and only known for low-dose MTX.

It might be conceived that a more optimal cDMARD treatment for PsA would be combination of optimally dosed MTX (25 mg) with LEF. Indeed, some evidence is available that combined MTX and LEF might be effective in the treatment of PsA, although large randomized clinical trials are lacking. A prospective observational study concluded that patients who were taking MTX and LEF combination therapy were more likely to achieve a PASI50 (i.e. 50% improvement on the Psoriatic Area Severity Index response) [14]. Another study found that a low-dose MTX and LEF regimen was effective in PsA [15]. In rheumatoid arthritis, the combination of MTX and LEF is one of the few cDMARD combinations for which additive effectiveness was suggested, with acceptable safety [16–18].

Therefore, the aim of our study is to investigate whether the effectiveness of optimally dosed MTX and LEF combination therapy is superior to optimally dosed MTX monotherapy in patients with active PsA. If MTX and LEF combination therapy is more effective and safe in PsA, this will provide a valuable low-cost treatment option for patients with PsA.

## Methods

This 16-week investigator-initiated single-center, randomized, placebo-controlled, double-blind clinical trial is currently (start inclusion: February 1, 2019) being carried out in patients with PsA at the departments of rheumatology of the Sint Maartenskliniek in the cities of Nijmegen and Woerden, the Netherlands. The recommended items to address in a clinical trial protocol (SPIRIT) checklist is shown in Additional file 1.

### Objectives

The aim of this study is to investigate whether the effectiveness of optimally dosed MTX and LEF combination therapy is superior compared to optimally dosed MTX monotherapy in DMARD-untreated patients with PsA with regard to disease activity and quality of life.

#### Primary objective

To assess the between-group difference in Psoriatic Arthritis Disease Activity Score (PASDAS) of MTX and LEF combination therapy versus MTX monotherapy in patients with PsA after 16 weeks, adjusted for baseline PASDAS.

#### Secondary objectives

Key secondary objectives that will be investigated (between treatment groups at week 16):

- The change in the different health domains of PsA (peripheral joint disease, enthesitis, dactylitis, axial involvement, and skin and nail psoriasis)
- The change in the Disease Activity in Psoriatic Arthritis (DAPSA) score
- The difference in proportion of patients meeting minimal disease activity (MDA) criteria, low disease activity (LDA) according to PASDAS (≤ 3.2) and DAPSA (≤ 14), and American College of Rheumatology (ACR) 20/50/70 response criteria
- The difference in quality of life
- The difference in adverse events (AEs)

### Study design

The COMPLETE-PsA trial is designed as a randomized placebo-controlled double-blind superiority trial. Seventy-eight DMARD-untreated patients with PsA will be randomized 1:1 to either the combination arm (MTX and LEF) or the monotherapy arm (MTX and placebo). To ensure equal allocation of patients with high disease activity (HDA), patients will be stratified in both groups by PASDAS ≥ 5.4 (= cutoff score for HDA) at baseline. This because regression to the mean is more likely to occur in patients with HDA and, from a biological point of view, treatment might be more effective in patients with HDA.

### Assessments

**Fig. 1:**
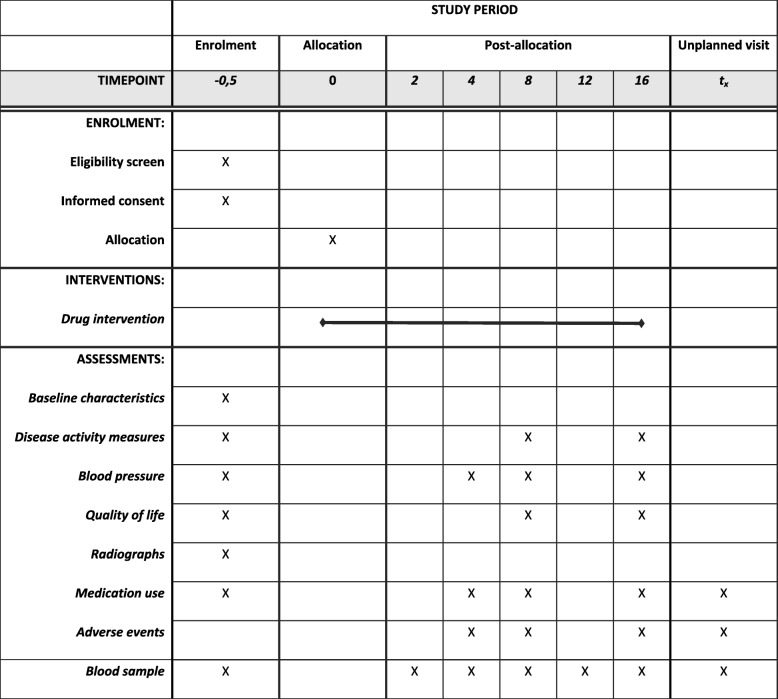
SPIRIT figure trial visits and assessments

Study visits are planned at baseline and week 8 and 16 (a 1-week window is permitted around scheduled study visits). The screening visit and baseline visit will be performed at the same time, to ensure timely start of treatment. Patients will be contacted by telephone at week 4 to screen for treatment intolerance. They will be asked whether they are experiencing any side effects, including high blood pressure, as measured by their general practitioner (GP) 2–4 weeks after start of medication. Patients will start with MTX 15 mg per week at week 0 either oral or subcutaneously, if this is well tolerated, the dosage will be increased to 25 mg per week at week 4 (after telephone consultation). Regular blood sampling will be performed to screen for toxicity. Within our study only baseline radiographs will be obtained, because differences between baseline and the endpoint (16 weeks) are not expected, as this period is too short to assess (progression of) structural joint damage on radiographs. In addition, this would also expose patients to unnecessary X-ray radiation. In Fig. 1, the study visits and assessments are described.

### Participants, intervention and outcomes

#### Inclusion criteria

1. Age ≥ 16 years.
2. Clinical diagnosis of PsA.
3. Evidence of active disease defined as ≥ 2 swollen joints, dactylitis counts as 1 swollen joint.
4. Patients who have used cDMARDs and/or bDMARDs before, must have discontinued this treatment for at least 6 months prior to baseline visit.
5. Patients who are already taking nonsteroidal anti-inflammatory drugs (NSAIDs)/ cyclooxygenase-2 (COX-2) inhibitors may participate in the study, but the dose has to be stable for at least 1 week prior to the first dose of study drug.
6. Intramuscular and intra-articular corticosteroids have to be discontinued or not given 8 weeks prior to the first dose of study drug. With the exception of a failed intra-articular corticosteroid injection (defined as remaining swelling and (if previously present) tenderness of the injected joint 2 weeks after the injection). In the case of a failed injection, patients can participate in the study 2 weeks after the intra-articular injection.
7. Oral corticosteroids have to be discontinued 10 days prior to the first dose of study drug.
8. If fumaric acid is used at baseline, this will be discontinued and switched to study medication (according to usual care and to prevent triple therapy).

#### Exclusion criteria

1. Female patients who are pregnant, breastfeeding or is considering becoming pregnant during the study or for approximately 2 years after the last dose of study drug or up to 11 days after treatment when washout procedure is executed.
2. Male patients who are considering fathering a child or donating sperm during the study or for approximately 2 years after the last dose of study drug or up to 11 days after treatment when washout procedure is executed.
3. History of an inadequate response to MTX or LEF (prescribed by a rheumatologist for joint disease).
4. Current severe infection including, but not limited to:
  - Active human immunodeficiency virus (HIV)
  - Active tuberculosis
5. History of an allergic reaction or significant sensitivity to constituents of the study drugs (MTX/LEF).
6. Current or history of hepatic disease, including, but not limited to:
  - Non-alcoholic fatty liver disease (NAFLD)
  - Non-alcoholic steatohepatitis (NASH)
  - Alcoholic cirrhosis
7. History of clinically significant (per investigator’s judgment) drug or alcohol abuse within the last 6 months prior to baseline visit.
8. Current or recent history of a severe, progressive, or uncontrolled renal, hematological, gastrointestinal, metabolic, endocrine, pulmonary, cardiovascular, or neurologic disease.
9. History of any fibromyalgia or diagnosis of inflammatory rheumatic disease other than PsA. Prior history of fibromyalgia is permitted if documentation of change in diagnosis to PsA or documentation that the diagnosis of fibromyalgia was made incorrectly.
10. Abnormal laboratory values within 1 month prior to baseline visit:
  - Serum alanine transaminase (ALT) > 1.5 × upper limit of normal (ULN)
  - Estimated glomerular filtration rate (GFR) by simplified four-variable Modification of Diet in Renal Disease (MDRD) formula < 40 ml/min/1.73 m2
  - Total white blood cell count (WBC) < 3000/μl
  - Platelet count < 100,000/μl
  - Hemoglobin < 10 g/dl (6.3 mmol/l)
11. Current persistent hypertension requiring start or change of treatment regimen.
12. Malignancy in the past 5 years except for non-melanoma skin cancer.

#### Patient recruitment

A total of 78 patients will be enrolled over a period of 30 months. All rheumatologists in our hospital will be informed about the study. Representatives of the study team will explain the nature of the study to the patient and will answer all questions regarding the study. Other rheumatologists in the Netherlands will be made aware of the study and are requested to recruit and refer eligible patients to the site involved in the study. During the regular visit at the outpatient clinic, an eligible patient will be briefly informed by his/her rheumatologist, receives a patient information letter (PIF), an informed consent form (ICF), and is asked for permission to be called by the coordinating investigator. The patient will be called within 3 days (to ensure the timely start of treatment) by the coordinating researcher, to ask whether the patient has any additional questions and agrees to participate.

### Intervention

#### Investigational product/treatment

Patients in the combination arm (MTX and LEF) will receive MTX 25 mg (oral or subcutaneous) once weekly plus two LEF 10 mg capsules daily. Patients in the monotherapy arm (MTX and placebo) will receive MTX 25 mg (oral or subcutaneous) once weekly plus two placebo tablets daily. We will perform a stepwise up-titration of MTX. In the first 4 weeks, the dosage will be 15 mg per week. At week 4, treatment tolerability and adverse events will be checked (telephone consultation). In the case the 15 mg per week dosage is well tolerated, the dosage will be increased to 25 mg per week. If the 15 mg per week dosage is not tolerated, it will not be increased. The total study duration is 16 weeks.

#### Use of co-medication

All patients will receive folic acid 10 mg/week during the study period to mitigate the side effects of MTX.

#### Follow-up treatment

After the end of study at 16 weeks, everyone (including the patient) is unblinded and patients will continue routine clinical care with their own physician. Further treatment decisions are per standard clinical care and based on shared decision-making. Shared clinical decisions between physicians and patients will allow patients to voluntarily stop or decrease the dosage on either MTX or LEF when stable and low disease activity is reached and the study has ended.

#### Escape treatment

In case of HDA per treating physician’s judgment, local topical psoriasis therapy, intra-articular corticosteroid injection(s) (after week 8, only one intra-articular injection is allowed), and alteration of type and dosage of NSAIDs are permitted during the entire study period. After baseline visit, intramuscular glucocorticoid injections are allowed until 8 weeks before the 16-week visit. Oral glucocorticoids are allowed until 10 days before the 16-week visit.

### Outcome measures

#### Primary outcome

The primary outcome is the difference between the combination therapy group (MTX and LEF) and the monotherapy group (MTX and placebo) on the PASDAS at week 16 adjusted for baseline PASDAS. The PASDAS is a disease-specific outcome measure, that was developed as part of a project that aimed to develop new composite measures for PsA, derived from real-world data (the GRACE project) [19]. It is a disease activity and comprehensive continuous outcome measure, taking many of the different health domains of PsA into account. Of note, the PASDAS received the highest number of votes for use in randomized clinical trials during a consensus meeting held by the GRAPPA and Outcome Measures in Rheumatology (OMERACT) [20].

The different components that are used for the PASDAS calculation are: a 66 swollen and 68 tender joint count (66/68 SJC/TJC), C-reactive protein (CRP), patient and physician global visual analogue scale (VAS), Leeds Enthesitis Index (LEI), dactylitis count and the physical component score (PCS) of the Survey Short Form-36 (SF-36). As research has shown that the PCS of the SF-36 can be substituted by the PCS of the SF-12, the SF-12 was chosen for use instead, despite the requirement of a license for its use [21]. A difference of 0.8 PASDAS points or more between treatment groups is considered to be clinically relevant, since 0.8 was found to be the PASDAS cutoff for response [22].

### Secondary outcome measures

#### Other disease activity outcome measures

As the best outcome measure for PsA in clinical trials remains under discussion, other disease activity outcome measures that are regularly used in PsA trials were also included. Disease-specific measures for PsA that have been proposed, in addition to the PASDAS, are the DAPSA and MDA criteria [23, 24]. The DAPSA is a continuous score that results from the summation of the 66 SJC, 68 TJC, patient global VAS, patient pain VAS and CRP. The MDA is a binary outcome measure, and is achieved when five of the seven following criteria are met: 66 SJC ≤ 1, 68 TJC ≤ 1, body surface area (BSA) ≤ 3, patient pain VAS ≤ 15, patient global VAS ≤ 20, health assessment questionnaire (HAQ) ≤ 0.5 and LEI ≤ 1. To be able to compare the study results with the results from other clinical trials, ACR 20/50/70 response was also measured, because the ACR criteria are well established and the most often used as a primary outcome in PsA trials to date.

#### Skin and nail scores

The BSA score and Physician Global Assessment (PGA) score will be measured at every study visit and the change in skin scores between groups will be assessed. The BSA and PGA were chosen because it is simpler to measure them compared to the Psoriasis Area and Severity Index (PASI). Furthermore, the product of the PGA and BSA (PGA×BSA) has shown to be a measure that is sensitive to change and that has a good correlation to the PASI [25]. The BSA score is measured by the percentage of skin that is affected and ranges from 0 to 100%. The PGA of psoriasis is measured on a nominal scale that ranges from 0 to 4 (0 = clear, 1 = almost clear, 2 = mild, 3 = moderate, 4 = severe) and measures plaque severity. Patients will be examined for nail psoriasis, and if nail disease is present, the ‘patient global VAS nail disease severity’ (0–100 mm) will be measured at every study visit.

#### Quality of life

For the measurement of quality of life, we use the SF-12, HAQ and the Psoriatic Arthritis Impact of Disease (PsAID) questionnaire. The SF-12 and HAQ are also used for calculation of the PASDAS and MDA criteria, respectively. The PsAID is a patient-reported outcome measure that was developed with the help of patient partners to measure the impact of PsA [26]. Research has shown that the PsAID is a reliable patient-reported outcome measure for the measurement of the impact of PsA and is sensitive to change [27]. Both the HAQ and the SF12 are validated non-disease-specific measures for functioning and health-related quality of life.

#### Safety

To assess if there is a difference in safety between the two treatment groups, all the (serious) adverse events (S)AEs will be tracked. At the end of the study, the percentage of (S)AEs will be compared between the two arms. As this study was deemed a low-risk study by the local ethics committee, no external data safety monitoring board was required. However, there is an internal and independent data safety monitoring committee that reviews protocol changes and data on safety and recruitment.

### Randomization, concealment, and blinding

Patients will be allocated to either MTX plus LEF (intervention) or MTX plus placebo (control) at baseline visit, stratified by PASDAS ≥ 5.4 (HDA). Patients will be allocated using stratified variable block randomization, to prevent predictability of allocation. Randomization is performed by a research physician or research nurse using a computerized randomization procedure. Allocation is kept in sealed and consecutively numbered envelopes. Patients, physicians, researchers, and nurses will be blinded for treatment allocation. The distribution and assignment of study medication, based on randomization number, is done by the hospital pharmacy. The placebo is manufactured compliant with Good Manufacturing Practice (GMP) guidelines and the placebo tablets are indistinguishable from LEF tablets. If needed, unblinding is possible after consulting the hospital pharmacist. All quantities that participants have taken (evaluated by pill count) are documented. Before the disposal of used, unused, and depleted pill boxed, a pill count will be performed.

### Sample size

The primary outcome for the study is the difference between the combination therapy group (MTX and LEF) and the monotherapy group (MTX and placebo) on the PASDAS at week 16. The sample size per arm for a *t* test having a power 1-β(=0.8) when testing at significance level α(=0.05) (one-sided) to detect a difference of δ is *N* = 2*(z_1-α_ + _z1-β_)^2^*SD^2^/δ^2^. Based on predefined response criteria for the PASDAS, the study was powered to detect a difference of δ = 0.8 [22]. The standard deviation of the PASDAS in PsA patients is reported to range from 1.31 to 1.63, we used the latter value in these calculation to be conservative [19]. One-sided testing was chosen because it is highly unlikely that a combination of MTX and LEF would be inferior (with regard to effectiveness) to MTX monotherapy. In addition, as it is not common practice to start with the combination therapy, the main focus of interest is to see if the combination is superior to methotrexate monotherapy (the first-line therapy to date). Furthermore, one-sided testing instead of two-sided testing leads to a reduction in patient numbers and costs. With these parameters, the required number of patients would be 52 per arm for a total of 104 patients. When correcting for baseline PASDAS score, this sample size can be reduced by (1-r^2^) where r is the correlation between baseline and follow-up. In addition, baseline PASDAS is a stratification factor, and therefore, should be adjusted for in the analysis. Based on information from the literature, the correlation between two PASDAS measurements is approximately 0.8 [22]. However, this correlation might be too optimistic, considering it is based on patients without changes in disease activity or treatment. Therefore, to protect for a too-optimistic correlation, a total trial size corresponding to a correlation of 0.5 was chosen. With this correlation of 0.5, a total number of 78 patients is required. The short follow-up of this trial will allow inclusion of additional patients should some drop out. No more than 10% (8 patients) dropout is expected, because of the short duration of the study, so the maximum number of patients to be included is 86 (43 patients per arm).

### Statistical analysis

All statistical analyses will be performed using STATA/IC 13.1 for Windows (StataCorp, College Station, TX, USA). Primary analysis will be based on intention-to-treat analysis. The primary endpoint will be tested using 90% confidence intervals with the PASDAS at week 16 as outcome, treatment group as determinant, and baseline values of PASDAS as covariate (ANCOVA). Missing values will be imputed where appropriate. Differences between groups on secondary outcomes will be tested with chi-square test (or Fisher exact test) for categorical variables (e.g., response criteria sets such as MDA and ACR 20/50/70) and with unpaired *t* test or non-parametric alternative (where appropriate) for continuous variables (e.g., DAPSA score, Quality of Life). Descriptive statistics will be provided using mean +/− SD, median (p25-p75) or percentages for primary and secondary outcomes, where appropriate.

### Data collection and monitoring

The collected data will be entered in CASTOR, an electronic database set up for clinical trials (https://www.castoredc.com/nl/waarom-Castor.html). Data will be coded and kept based on the rules for good clinical practice (GCP) by certified personnel. Handling of personal data will comply with the Dutch Personal Data Protection Act (WBP, wetbescherming persoonsgegevens). Data of all centers will be monitored following the guidelines of the St Maartenskliniek. Data will be stored for 20 years after the end of the study. Patients will be asked for permission to use their data (anonymously) for additional research in the field of PsA, as described in the patient information brochure. An independent monitor will be appointed to monitor the study according to the monitoring plan. Monitoring and quality assurance will be performed according to the advice of the NFU (Dutch Federation of University Medical Centres).

#### Ethical consideration

This study has received ethical review board approval from the central Commissie Mensgebonden Onderzoek (CMO) regio Arnhem Nijmegen, Radboud University Medical Centre (number NL66544.091.18), dated November 19, 2018. It is registered in the Dutch Trial Register, NTR 7632, dated December 3, 2018. Important protocol modifications will be submitted for review to the ethics committee and communicated to the trial register. Informed consent will be obtained from all the participants. If the patient is interested, the baseline visit will start with signing the ICF by both the patient and coordinating researcher. Before signing, the ICF needs to be fully understood and there will be a possibility for the participant to ask any additional questions. The study will be performed in accordance with the ICH Good Clinical Practice (GCP) guidelines and all relevant legislation.

## Discussion

PsA is a disabling disease with a negative impact on quality of life. However, the effectiveness of cDMARDs, that are most often prescribed as first-choice treatment in PsA, remains unclear due to a lack of high-quality trials. This is because in the past PsA trials were considered less urgent due to the impression that PsA is a rather mild disease compared to RA, and the lack of validated classification criteria and outcome assessments. Factors hampering the execution of PsA trials are the lower incidence compared to RA, hampering sufficient patient inclusion, and the heterogeneity of the disease complicating outcome assessment. In addition, trials with cDMARDs are in general less attractive to perform due to a lack of financial support by pharmaceutical companies.

Noteworthy, MTX has been used as the first-choice treatment option in PsA for many years, although its effectiveness, in line with the effectiveness of other cDMARDs, has not been well established and seems to be slightly disappointing. The efficacy of MTX in psoriasis and RA may have added to impression that MTX is an effective treatment for PsA. In this study, the aim is to assess if a combination of MTX and LEF might be a better option as a first-line treatment regimen than optimally dosed MTX monotherapy. If the combination indeed proves to be effective and of added value, this may lead to a reduction in bDMARD use and thereby infection risk and costs. This may be of interest especially to low-income countries, where bDMARD availability is limited.

During the development of this research protocol, there were different issues that needed to be addressed and choices that had to be made. To start, a primary outcome measure for this trial had to be chosen. So far, it is not clear what the most optimal outcome measure for clinical trials in PsA is. The optimal outcome measure would preferably be a continuous measure, that is disease-specific and includes the different domains of the disease. Furthermore, it has to be reliable and sensitive to change. For this trial, different outcome measures were considered, including: the DAPSA, the MDA criteria, the Composite Psoriatic Disease Activity Index (CPDAI), the ACR 20/50/70 response criteria and the PASDAS. Although the DAPSA is easy to calculate, it has unfavorable clinimetric properties. It is not parametrically distributed, has a large measurement error, does only take the peripheral joint disease domain into account, and it was unable to discriminate between treatment groups in two different studies, in contrast to ACR responses and MDA measurement [28, 29]. The MDA criteria result in a dichotomized outcome (fulfillment of the criteria, yes or no) and consequently the need of a larger sample size than would be needed with a continuous outcome measure. The CPDAI is a continuous outcome measure, but has less discriminative capacity than other outcome measures [30]. The ACR response criteria are not disease-specific, are dichotomous, and do not include all the PsA disease domains. Contrarily, the PASDAS is a disease-specific, continuous, reliable and valid outcome measure that includes most of the disease domains of PsA. The use of the PASDAS as a primary outcome measure is further enabled by the integration of routine and standardized PASDAS measurements in routine clinical practice [31]. Based on these considerations, the PASDAS was chosen as the primary outcome measure for this trial.

Second, decisions about the design of the study had to be made. Although the effectiveness of MTX monotherapy remains not fully clear, especially the 25 mg dose, a trial comparing MTX versus placebo was deemed unethical, as most of these patients have already failed to respond to treatment with NSAIDs and/or intra-articular injections, and MTX is a widely accepted first-line treatment according to EULAR and GRAPPA guidelines. This would have made patient inclusion not feasible. Instead, using MTX monotherapy as an active control condition, the first-choice in the field, will provide us with information that directly translates to routine clinical practice. If MTX and LEF combination therapy is indeed more effective than MTX monotherapy, this will result in faster remission induction and, possibly, improvement in prognosis [32]. Currently, the combination of MTX and LEF is also being studied as the starting treatment arm of a large PsA treatment strategy study, the SPEED study [33]. In addition to using MTX monotherapy as the control condition, a LEF monotherapy control condition would also be an option, since the effectiveness of LEF monotherapy is not well-established either. However, adding an extra arm to this trial would result in a much larger sample size that would severely hamper its feasibility. In the case MTX and LEF combination therapy is superior, conducting an additional study that compares MTX and LEF combination therapy with LEF monotherapy will be considered.

Third, the dosage of MTX and LEF and the administration route for MTX had to be chosen. In line with the evidence-based recommendations for the use of MTX in rheumatic diseases, oral MTX and folic acid 10 mg/week with a MTX start dose of 15 mg/week that is increased, if well tolerated, to 25 mg/week after week 4, was chosen [34]. In case of intolerance, MTX tablets may be switched to MTX subcutaneous injections. In addition to intolerance, patients with a strong preference for parenteral administration are allowed to start with subcutaneous injections. MTX is used in both arms and thus is open-label, so no placebo tablets or injections for MTX had to be manufactured. In contrast to MTX, LEF is only available in oral form in 10 and 20 mg tablets. Previous research in RA has shown that, paradoxically, (S)AEs resulting in treatment discontinuation were higher when LEF 10 mg/day was used compared with LEF 20 mg/day, whereas efficacy was lower in the 10 mg/day group compared with the 20 mg/day group [35]. For this reason, LEF 20 mg/day will be used in this study. Although 20 mg tablets are available, 10 mg tablets were chosen. Every patient receives two LEF tablets or two placebo tablets daily. This enables dose reduction of LEF from 20 to 10 mg if needed, without unblinding of treatment allocation. Safety will be monitored closely and, according to the local toxicity protocol and MTX recommendations, lab testing will be done every 2 weeks the first month followed by every 4 weeks thereafter until conclusion of the study to check for any blood abnormalities [34]. Although in studies in RA the combination of MTX and LEF did not result in more laboratory abnormalities than MTX alone, it is possible that PsA patients are not completely comparable with RA patients with regard to side effects [17, 18].

Last, the permission of glucocorticoid use in the period before baseline visit had to be determined. Patients sometimes receive glucocorticoids before they are referred to a rheumatologist from their GP, have received local (intra-articular) treatment before they start with cDMARD treatment, or have very active disease that demands the use of glucocorticoids in the obligated waiting time between receiving patient information and baseline visit. But the use of glucocorticoids just before baseline visit might influence the baseline measurements. Accordingly, a good balance between external generalizability and minimizing bias of the baseline measurements is needed. Oral corticosteroids have to be discontinued 10 days prior to baseline visit, to ensure the biological effect has faded. This is based on the general principle that after five times the half-life of a drug (36 hours for oral glucocorticoids), 97% of the ingested dose is removed from the body [36]. This results in a washout period of 7.5 days. To assure the return of patients’ immune system to the basal state, this period was extended by 33% to 10 days. Since intramuscular and intra-articular corticosteroids have a longer presence and (residual) activity than oral corticosteroids, they have to be discontinued 8 weeks prior to inclusion. With the exception of a failed intra-articular corticosteroid injection (defined as remaining swelling and [if previously present] tenderness of the injected joint 2 weeks after the injection). In the case of a failed injection, patients can participate in the study 2 weeks after the intra-articular injection. Since this will not result in disturbance of the baseline SJC/TJC and will also contribute to external generalizability.

In conclusion, this investigator-initiated study will provide essential additional information on the effectiveness of MTX and LEF combination cDMARD therapy in PsA It will provide important information for treatment strategies and treatment recommendations in PsA.

### Trial status

Open for inclusion. Recruitment started in February 2019 and will likely be completed in September 2021.

Protocol version: 1.3. October 1, 2019.

## Supplementary information

**Additional file 1.** SPIRIT 2013 Checklist: Recommended items to address in a clinical trial protocol and related documents.

## Data Availability

The datasets used and analysed during the current study are available from the corresponding author on reasonable request.

## Abbreviations

ACR: American College of Rheumatology
AE: Adverse event
bDMARD: Biological disease-modifying antirheumatic drug
BSA: Body surface area
cDMARD: Conventional disease-modifying antirheumatic drug
CRP: C-reactive protein
DAPSA: Disease Activity in Psoriatic Arthritis score
EMA: European Medicines Agency
EULAR: European League Against Rheumatism
GP: General practitioner
GRAPPA: Group for Research and Assessment of Psoriasis and Psoriatic Arthritis
HAQ: Health Assessment Questionnaire
HDA: High disease activity
LDA: Low disease activity
LEF: Leflunomide
LEI: Leeds Enthesitis Index
MDA: Minimal disease activity
MTX: Methotrexate
NSAID: Nonsteroidal anti-inflammatory drug
PASDAS: Psoriatic Arthritis Disease Activity Score
PASI: Psoriatic Area Severity Index Response
PCS: Physical component score
PGA: Physician Global Assessment
PsA: Psoriatic arthritis
PSAID: Psoriatic Arthritis Impact of Disease
SAE: Serious adverse event
SF-12: Short Form 12
SJC: Swollen joint count
TJC: Tender joint count
TNFi: Tumor necrosis factor alpha inhibitor
VAS: Visual Analogue Scale
